# Miscellanies

**Published:** 1843-07-01

**Authors:** 


### Miscellanies.

---

QUARTERLY TABLE OF THE MORTALITY IN 114 OF THE PRINCIPAL TOWN OR CITY DISTRICTS OF ENGLAND. Winter Quarter of 1843, ending 31st March.

This quarterly return is derived from 114 districts, the enumerated population of which at the last census was 6,534,535, or nearly *four-tenths* of the total population. The average annual number of deaths registered in the 114 districts was 163,193, or 47 per cent. of the total deaths registered annually in England. Taking the averages of the five last years, it appears that the mortality attained its maximum in the year 1840, and was the lowest in the year 1842. Out of 100,000 persons living in the towns and cities of England, about 2658 died in the year 1840, and only 2420 in the year 1842.

The number of deaths which occurred in the Winter quarter 1843, was 43,466; though, according to the average of the last five years, it ought to have been 47,542.

In the *Metropolis* the mortality is still less than the average, amounting to 12,312, the population in 1841 being 1,870,727.

With regard to the diseases which have prevailed during the quarter, we find that the deaths by epidemic diseases (2071) were 363 less than the average of the five preceding Winters. The deaths from *typhus* have however increased from about 30 to 50 weekly. *Small-pox, hooping-cough, influenza,* and *erysipelas,* have been less fatal than in the five preceding Winters. No case of death from *hydrophobia* occurred, and only 3 from *tetanus,* 456 from *asthma,* 1 from *intussusception,* 54 from *mortification,* 267 from *violent death.* Of dysentery (33), cholera (6), tabes mesenterica (73), jaundice (34), disease of liver (117), and ovarian dropsy (10)—a greater number than is usual—died in the metropolis.

The epidemic diseases most frequently mentioned by the registrars in the country are, *typhus, scarlatina,* and *hooping-cough.*

Some districts, in which the number of deaths was *above* the average of the Winter quarters (1838—42) in the same districts:—Brighton, Bedford, Yarmouth, Devizes, Plymouth, Penzance, Stroud, Hereford, Woolstanton and Burslem, Coventry, Basford, Macclesfield, Blackburn, Huddersfield, Newcastle-on-Tyne.

Some districts, in which the number of deaths was *below* the average of the Winter quarters in the same districts:—Windsor, Oxford, Cambridge, Ipswich, Norwich, Bath, Bristol, Clifton, Worcester, Kidderminster, Dudley, Wolverhampton, Birmingham, Liverpool, Preston, Bury, Wigan, Manchester, Sheffield, Bradford, Hull, Sunderland, Carlisle, Kendal, Merthyr Tydvil.

*Meteorology.*—The average height of the barometer was 29.715 inches, or 0.134 parts of an inch lower than the average. The mean temperature was 1.3 above the average. The fall of rain in the quarter was 3.429 inches in 29 days, while the Winter average is 2.886 inches in 26 days. The South and West winds prevailed.

---

DIRECTIONS TO THE REGISTRARS OF DEATHS.

[*To the Editor of the Medico-Chirurgical Review.*]

General Register Office, 6th May, 1843.

SIR,—I shall feel much obliged if you will inform the readers of the *Medico-*

*Chirurgical Review*, that the Registrar General has recently given the following directions to the Registrars of Deaths.

"When the information given to you by the medical attendants upon deceased persons, respecting the causes of death, contains also a statement of the *duration of the fatal diseases*, or is accompanied by the memorandum '(p. mort.)' denoting that the nature of the causes of death had been ascertained or verified by a *post-mortem* examination; you will not fail to enter these statements in the column of the Register headed 'cause of death.'"

It is desirable on many accounts that the laws respecting the duration of diseases should be ascertained, and this will afford the medical profession an opportunity of entering on permanent records a sufficient number of observations for determining those laws, as well as the laws of mortality.

With regard to the registration of the "causes of death," it has been found the most convenient and satisfactory course, for the medical attendant to leave a written certificate, with the friends of the deceased person,—to be placed in the hands of the Registrar.

The Registrar is directed to ask the informant whether any written statement of the cause of death has been left by the medical attendant, but the relatives from the natural distraction of grief,—or ignorance of its scientific importance,—are apt to forget the medical certificate unless the medical attendant take the trouble to state that it will be required, and place it in their hands.

If the medical profession needed any stimulus to induce them to contribute to the promotion of medical science, or to the discovery, and consequent removal of the causes of untimely death, it will be found in the following considerations to which the Registrar General has adverted in his last Report.

"I hope the members of the medical profession, who have hitherto given their aid, will cordially assist in carrying out this national registration of the causes of death, as they alone are able to give a correct statement of the nature of the fatal diseases; and to them more than to the members of any other profession, must be apparent the vast importance of thus collecting accurate materials for advancing the science of Vital Statistics."

Copies of the Statistical Nosology with notes and observations for the use of those who return the "causes of death,"—may be procured by any medical practitioner upon application (verbal or written) at the General Register Office.

I have the honor to be,

Sir,

Your obedient servant,

WILLIAM FARR.

---

### A New kind of Pessary.

Mr. Snow, has invented a new kind of Pessary, which consists of a piece of sponge cut into the form of a sphere, and tied up, by means of small twine or silk thread, in a circular piece of oil-skin, in such a manner that a small stem or tail, about half or three-quarters of an inch long, is left. The firmer kinds of sponge are to be preferred, and the oiled silk ought to be closed as firmly as possible by tying.

On compressing this pessary, the air in the cells of the sponge is gradually forced out at the neck, between the folds of the oil-skin, when it can be very readily introduced. When passed above the contracted part of the vagina, air soon re-enters the cells, and the instrument becomes expanded. The tail facilitates its removal at any time.

The advantages which Mr. Snow considers this pessary to possess, are—

1. Its capability of being diminished in size during its introduction and removal.

2. Its softness, which is such that it can scarcely cause any of the effects of a foreign body.

3. Its small weight.

4. The tendency of its elasticity to keep it in its position, for any sudden pressure of the viscera above will be spent in overcoming this elasticity, instead of forcing the instrument through the external parts, and such pressure will flatten it and make it wider, thus rendering its extrusion the less possible.

It also possesses the advantages of being cheap and durable.

---

### Should the Child be placed to the Mother's Breast shortly after Delivery?

Such is the question asked by Dr. Hocken, and answered thus.

Both the mother and the child are benefitted by the early application of the latter to the former.

1st. It leads to contraction of the uterus and prevents hæmorrhage. Such an occurrence never takes place when once the mother has given suck to her own child; no uneasiness need be entertained on this point if we have seen the child applied to the mamma before leaving the patient's house, for under these circumstances, the uterus is sure to maintain a firmly contracted condition. Besides, the contraction of the womb obviates the formation of clots, which subsequently give rise to painful efforts for their dislodgement.

"We thus avoid, by placing the child to the breast early, continued and severe after-pains, and what is in some cases of much greater importance, a profuse, continued, and sanguineous discharge of the lochia. Firm clots of blood will sometimes remain for some time, and undergo putrefactive changes in the uterus, and I think that there can be little doubt that absorption of putrefactive matters into veins, and the general circulation, does occasionally happen from such causes. No one can doubt that such causes would prove adequate to occasion phlebitis or the worst forms of malignant fever, or that having such an origin, it should not be capable of secreting a morbid poison, capable itself of propagating the disease to others."

2. The measure tends to suppress hæmorrhage which has occurred. "Even," says Dr. H. "in those severe and alarming states, where restlessness and jactitation have already set in, where one fainting fit succeeds another, the skin is covered with a cold and profuse perspiration, and the breath feels cold and damp, placing the child to the mother's breast will never fail, provided the woman is capable of recognising her offspring. The countenance brightens, a gush of blood and coagula show that the womb has expelled its contents, and by its firm contraction resists future hæmorrhage. The mere act of sucking the breast is not of itself sufficient; for these favourable results will not follow if another child be placed to the breast, or if the mother be incapable of recognising her own offspring."

3. At the time of the child's birth, the nipple projects freely, and is easily seized by the child. If, however, the child be not placed at the breast for a day or two, it becomes considerably enlarged from the accumulated secretion of milk, the nipple becomes buried instead of projecting, and the child experiences the greatest difficulty in seizing it. In this process the nipple suffers considerable violence, and the thin delicate skin covering it is apt to crack, and form painful fissures, by the child's constant attempts, which are kept up and increased by the continued difficulty.

The prevention of accumulation, too, in the milk ducts obviates mammary inflammation and abscess.

4. "Immediately the child is born, and sometimes even before this, it will instinctively suck anything introduced into its mouth, and seek the nipple when placed to the breast. On all hands it is allowed that a gentle aperient is requisite to get rid of the accumulated meconium; but why have recourse to substances which will disagree with the *primæ viæ*, when Nature has provided a most beautiful and simple aperient medicine in the colostrum, or the first formed serous milk? The child is born hungry, and instinctively desirous of the nipple, but instead of gratifying its natural taste, a quantity of artificial and, to it, indigestible food and irritating medicines, are thrust down its throat."

In all this there is much good sense, and it is difficult, on the ground of reason or common sense, to argue that nature intended a child not to suck it's mother's breast for 24 hours, or be crammed with castor oil as soon as it is born.

---

### Cure of Venereal Warts.

In a late Number of the Lancet Mr. Francis states that two remedies which he has tried for the extirpation of venereal warts, have always perfectly eradicated them, namely powdered savine and a solution of lunar caustic; the first to be applied to the warts every night, taking care previously to wet them, in order that the powder may adhere to them. The quantity ought not to be more than will lie on the top of a good-sized horse-bean. Applied every night for a week or ten days, this remedy will, it is said, cure them effectually. Should this, however, not be considered powerful enough, the savine may be sprinkled every night, and on the following morning a solution of nitrate of silver (four grains to the ounce) may be applied. These two remedies Mr. Francis always employs, and has never found them useless.

---

### Society for Relief of Widows and Orphans of Medical Men in London and its Vicinity.

The annual dinner of this Society took place on Saturday, June 3rd, at the Freemasons' Tavern, and was attended by about seventy of the members and their friends. The Duke of Cambridge, who presided, announced that he had accepted the office of Patron, and would at all times willingly promote the Society's welfare.

Donations were announced to the amount of £.308 15s., being a much larger sum than usual.

---

### Westminster Hospital.

On the 6th of June, Mr. Guthrie resigned the office of surgeon to this Institution; he will still, however, afford his services as consulting surgeon, and will deliver annually a course of clinical lectures. Mr. Hale Thomson has succeeded to the appointment of surgeon, and Mr. C. Guthrie to that of assistant-surgeon.

---

### St. Thomas's Hospital.

Mr. M'Murdo has succeeded Mr. Tyrrell as surgeon to St. Thomas's Hospital.

---

### GUY'S HOSPITAL.

In consequence of the state of his health, Dr. Bright has resigned the office of senior physician to Guy's Hospital, and has been succeeded by Dr. Addison. Drs. Barlow and Rees are made physicians to the Hospital, and Dr. Golding Bird assistant-physician.

